# Acute aseptic meningitis as the initial presentation of a macroprolactinoma

**DOI:** 10.1186/1756-0500-7-9

**Published:** 2014-01-07

**Authors:** Marina Boscolo, Danielle Baleriaux, Nathalie Bakoto, Bernard Corvilain, France Devuyst

**Affiliations:** 1 Department of Endocrinology, Erasme Hospital, Route de Lennik 808, 1070 Brussels, Belgium; 2 Department of Radiology, Erasme Hospital, Route de Lennik 808, 1070 Brussels, Belgium; 3 Department of Endocrinology, CHU Saint Pierre, Rue Haute 322, 1000 Brussels, Belgium

**Keywords:** Macroprolactinoma, Meningitis, Pituitary apoplexy, Rhinorrhea

## Abstract

**Background:**

Meningitis is an uncommon complication of an untreated pituitary macroadenoma. Meningitis may occur in patients with macroadenomas who have undergone transsphenoidal surgery and radiotherapy and is usually associated with rhinorrhea. Less commonly, cerebrospinal fluid rhinorrhea has been reported as a complication of treatment of prolactinomas by dopamine agonists. Cerebrospinal fluid rhinorrhea in cases of untreated pituitary macroadenoma is reported only in isolated cases. Acute bacterial meningitis without rhinorrhea in patients with an untreated pituitary macroadenoma is an exceptional finding with only three previously reported cases.

**Case presentation:**

A 31-year-old male was urgently admitted for headache, fever and visual loss. Neuroimaging disclosed an invasive pituitary lesion. Cerebrospinal fluid leakage was not clinically detected. Lumbar puncture showed acute meningitis. Blood tests revealed increased inflammatory markers, a serum prolactin of 9000 ng/ml (2.5-11 ng/ml) and panhypopituitarism. Intravenous antibiotics and hydrocortisone replacement therapy were initiated, leading to a favorable clinical outcome. An endoscopic transsphenoidal debulking procedure was performed, it showed that the sphenoid floor was destroyed and the sinus occluded by a massive tumor.

**Conclusions:**

Meningitis should be ruled out in patients with a pituitary mass who present with headache and increased inflammatory tests, even in the absence of rhinorrhea.

## Background

Typical signs leading to the diagnosis of invasive macroadenoma are visual impairment, hypogonadism and rarely signs of intracranial hypertension. Meningitis is an unusual first clinical manifestation of an invasive pituitary adenoma. A missed or delayed diagnosis could have a major impact on morbidity and mortality. Meningitis in a patient with an invasive pituitary macroadenoma is generally due to an infection of cerebrospinal fluid (CSF) leaking through the disrupted bony skull into the sphenoid sinus, allowing the entry of nasopharyngeal organisms [1,2]. CSF leakage in an uninfected patient commonly manifests as clear rhinorrhea. This is an uncommon but well known complication of transsphenoidal surgery and radiotherapy. Less frequently, dopamine agonists can induce CSF rhinorrhea in prolactin-secreting pituitary adenomas due to the abrupt shrinkage of the tumor [3-5]. This complication is generally reported within a few weeks or up to two years after the beginning of treatment [3,6].

CSF rhinorrhea in an untreated pituitary macroadenoma has been reported only in isolated cases. We report a case of an untreated invasive macroprolactinoma revealed by acute meningitis. The diagnostic and therapeutic challenges of bacterial meningitis in case of an invasive pituitary adenoma are discussed.

## Case presentation

A 31-year-old man presented at the Emergency Department with a six day history of severe headache and vomiting. No history of trauma or neurosurgery was reported. The patient mentioned an important visual loss in the left eye and a decreased libido for at least two years. No history of rhinorrhea was reported.

**Figure 1:**
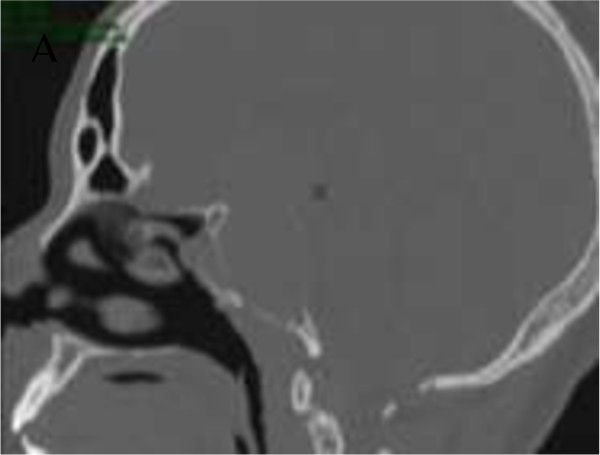
Sagittal CT image of the pituitary macroadenoma showing the bony erosion of the sellar floor and of the anterior wall of the sphenoid sinus.

**Figure 2:**
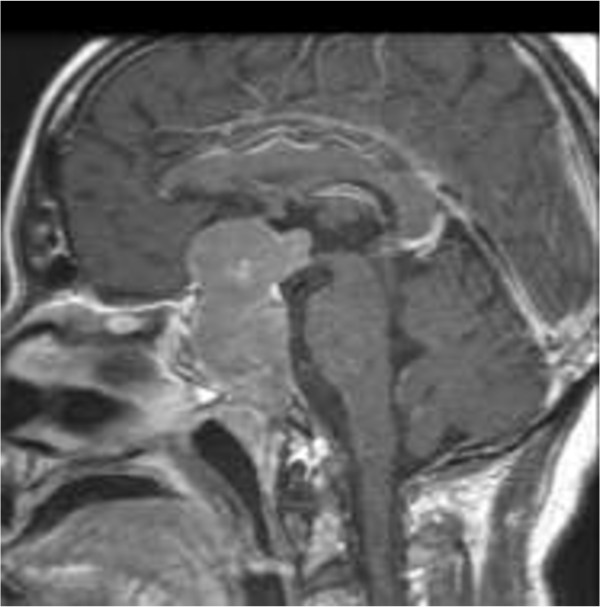
Sagittal Gadolinium-enhanced MRI of the large and invasive macroadenoma, with supra- and infra-sellar extension.

On admission, clinical examination revealed a normal level of consciousness. Complete left eye blindness with no deficit of other cranial nerves was observed. No rhinorrhea was detected. Body temperature was of 38°C. Computerized tomography (CT) and magnetic resonance imaging (MRI) disclosed a large, invasive pituitary lesion, which extended to the cavernous sinuses, to the sphenoid sinus and to the third ventricle, compressing the optic chiasm (Figure 1, Figure 2). No intratumoral hemorrhage was observed. Through CT scan an extensive disruption of the sellar floor with tumor extension within the sphenoid sinus were observed. Laboratory tests revealed an elevated C-reactive protein (305 mg/L, N <10 mg/L), a white blood cell count of 13,000/μl (4,000-10,000/μl) a mild hyponatremia (131 mEq/L, N 135–145 mEq/L), with a glycaemia of 127 mg/dl (70–100 mg/dl). Prolactin level was 9000 ng/ml (2.5-11 ng/ml); the other hormone measurements were suggestive of an associated panhypopituitarism (Table 1). At lumbar puncture, a purulent CSF was obtained, containing 11,900 white blood cells/μl (0-5/μl), 2680 red blood cells/μl, 6.3 g/L proteins (0.15-0.45 g/L), 39 mg/dl glucose (45–80 mg/dl) and 81 mg/dl lactate (10–22 mg/dl). These findings were consistent with a diagnosis of meningitis associated with a macroprolactinoma. Ceftriaxone was administered intravenously at the dose of 2 g × 2/24 h for 14 days, with favorable clinical evolution and normalization of inflammatory tests. CSF Gram stain and culture was negative for bacteria. Cryptococcal antigen testing was negative. Culture and polymerase chain reaction assay for detection of Mycobacterium tuberculosis were negative. Polymerase chain reaction for detection of viruses was also negative. A replacement therapy with hydrocortisone was initiated, alongside a treatment with cabergoline at the dose of 0.5 mg twice a week. A replacement therapy by levothyroxine at progressive doses was also administered.

**Table 1:** Serum pituitary hormone levels, measured at admission and at 1-year follow up

**Hormone levels**	**At admission**	**At 1-year follow up**	**Normal values**
Prolactin (ng/ml)	9000	6	2.5–11
Cortisol (μg/dl)	1.3	2.5	7–25
ACTH (pg/ml)	1.5	37	10–80
Total testosterone (ng/dl)	3	252	400–1200
FSH (mUI/ml)	0.5	-	1.5–12
LH (mU/ml)	<0.2	-	1–8
TSH (μUI/ml)	0.35	0.24	0.3–4
FT4 (ng/dl)	0.6	1.2	0.8–2
IGF-1 (ng/dl)	29	60	100–459

At endoscopic transsphenoidal approach a large bony defect in the inferior part of the sphenoid sinus was detected, obstructed by a massive tumor protrusion. Intraoperatively, a dural defect was not clearly identified. A significant reduction in tumor size was achieved through surgery, confirmed by MRI imaging. Histopathology confirmed the diagnosis of pituitary adenoma. No evidence of hemorrhage or necrosis was detected. No recovery from visual loss and panhypopituitarism was observed. Treatment with cabergoline 0.5 mg twice a week and levothyroxine 75 μg per day was continued, with normalization of serum prolactin and free T4 (Table 1). Hydrocortisone at a dose of 30 mg per day was also pursued as a follow up treatment.

## Discussion

The incidence of meningitis in patients with untreated pituitary macroadenomas is low and difficult to be estimated, as most information comes from case reports or small series [3,7]. Meningitis represents a well known risk for patients with CSF rhinorrhea appearing after a transsphenoidal surgical approach or radiotherapy. Less frequently, CSF rhinorrhea may be induced by dopamine agonists, as a consequence of the shrinkage of a medically treated macroprolactinoma. Exceptionally, CSF rhinorrhea may occur in untreated pituitary macroadenomas [4,5].

The first study assessing the incidence of nonsurgical CSF rhinorrhea among patients with pituitary adenomas was published in 2007 by Suliman et al. [7]. A large series of 114 patients with an invasive macroprolactinoma was compared to 181 patients with non-functioning pituitary macroadenoma. Among the 114 subjects presenting with macroprolactinoma, ten patients presented CSF rhinorrhea. Seven of these cases were dopamine agonist induced and three of them experienced spontaneous rhinorrhea. In two out of the three subjects with spontaneous rhinorrhea bacterial meningitis was the initial presentation. Within the group with spontaneous rhinorrhea, there was a clear male preponderance possibly related to a more aggressive behavior of prolactinoma in men. Spontaneous rhinorrhea was not observed in patients with non-functioning macroadenomas. Twenty other cases of CSF leakage in patients with untreated pituitary macroadenomas have been reported. Eight developed bacterial meningitis, mostly due to *Streptococcus pneumoniae*[2,5].

Our patient presented with acute bacterial meningitis in an untreated pituitary macroadenoma not preceded by CSF leakage. Only three cases have been reported in the literature. Their major clinical characteristics are summarized in Table 2. In two cases hormonal studies were suggestive of a macroprolactinoma, whereas in one case it was a non-functioning pituitary macroadenoma. A fistula with CSF leakage was identified on neuroimaging in two cases. A favorable outcome was observed in two cases. A late diagnosis of meningoencephalitis with subsequent mortality was described in one case [2,8,9]. The reported cases highlight the usefulness of a complete neuroimaging study and CSF analysis, even when no previous dopamine agonist treatment or rhinorrhea is reported. On the other hand, in cases of acute inflammatory features and a known invasive macroadenoma, differential diagnosis between bacterial meningitis and pituitary tumor apoplexy may be clinically difficult to establish. Pituitary apoplexy is the most frequent acute complication of macroadenoma. It is a result of a sudden increase in intrasellar pressure due to intratumoral hemorrhage. Severe headache, visual disturbances and signs of meningeal irritation are some of the clinical signs present both in bacterial meningitis and pituitary apoplexy [10]. Although CSF analysis is normal in most cases of apoplexy, if necrotic tissue has penetrated into the subarachnoid space it may show pleiocytosis, high protein levels and low glucose concentration [10,11]. Whilst bacterial meningitis is an exceptional event, tumor apoplexy is a well known complication of pituitary macroadenomas [10,12]. Its real prevalence is difficult to establish. In a study of 664 patients surgically treated for pituitary adenomas, typical symptomatic pituitary apoplexy was observed in 0.6% of patients but hemorrhagic and necrotic changes were seen in 9.6% of surgical specimens [6]. A review by Nawar et al. reported a rate of pituitary apoplexy between 12 and 25% in patients with a previous diagnosis of pituitary macroadenoma [10]. In our case, no sign of pituitary apoplexy was detected by CT and MRI.

**Table 2:** Clinical characteristics of the three reported cases of meningitis without prior history of CSF rhinorrhea with untreated pituitary macroadenomas

**Authors**	**Utsuki, 2004**	**Honegger, 2009**	**Robert, 2010**
Sex	Male	Male	Female
Age	69 years old	64 years old	32 years old
Hormonal secretion	Prolactin	Prolactin	Non-functioning
CSF culture	*S. pneumoniae*	Negative	*S. pneumoniae*
Blood cultures	Not reported	*S. pneumoniae*	Not reported
CSF fistula on neuroimaging	None	Yes	Yes
Surgery	Transsphenoidal debulking	Transsphenoidal debulking and leak repair	Decompressive craniotomy
Outcome	Favorable	Favorable	Death

## Conclusions

Meningitis is an unusual complication of untreated invasive pituitary adenomas and only represents the initial symptom leading to diagnosis of a macroadenoma in exceptional cases. Differential diagnosis between meningitis and pituitary apoplexy in a patient with an invasive macroadenoma presenting with headache and fever may be challenging. In the presence of inflammatory biology, a lumbar puncture must be performed without delay to treat rapidly meningeal infection. A missed or delayed diagnosis could have a major impact on morbidity and mortality. The absence of CSF rhinorrhea does not rule out the possibility of meningitis.

## Consent

Written informed consent was obtained from the patient for publication of this case report and any accompanying images. A copy of the written consent is available for review by the Editor of this journal.

## Abbreviations

CSF: Cerebrospinal fluid; CT: Computerized tomography; MRI: Magnetic resonance imaging.

## Competing interests

The authors declare that they have no competing interests.

## Authors’ contributions

MB led the conception of this case report, performed the review of literature and drafted the manuscript. NB provided clinical care for the patient. DB performed radiologic interpretation. BC and FD critically reviewed the manuscript. All authors read and approved the manuscript.
